# Supplementary material for: CF-Seq, an accessible web application for rapid re-analysis of cystic fibrosis pathogen RNA sequencing studies
Source: Sci Data. 2022 Jun 16;9:343. doi: 10.1038/s41597-022-01431-1 (PMC9203545; doi:10.1038/s41597-022-01431-1)
Supplement: Supplementary file 1 — Supplementary Information [file 41597_2022_1431_MOESM1_ESM.pdf]

CF-Seq, an accessible web application for rapid re-analysis of cystic fibrosis pathogen RNA sequencing studies

Supplementary Information

Contents

Table S1. Clinical relevance of CF pathogens included in the application
Table S2. Review of similar applications
Table S3. Study Data Acknowledgements
Table S4. Example Table of Differentially Expressed Genes
Table S5. Beta Testing Criteria – Filtering Studies
Table S6. Beta Testing Criteria – Viewing Study Metadata
Table S7. Beta Testing Criteria – Running Analysis

Figure S1. Zoomed-in image of figure 2 (application workflow), panel 1 (user manual)
Figure S2. Zoomed-in image of figure 2 (application workflow), panel 2 (study view)
Figure S3. Zoomed-in image of figure 2 (application workflow), panel 3 (filter studies)
Figure S4. Zoomed-in image of figure 2 (application workflow), panel 4 (additional metadata)
Figure S5. Zoomed-in image of figure 2 (application workflow), panel 5 (analysis view)
Figure S6. Zoomed-in image of figure 2 (application workflow), panel 6 (visualize pathway)

Supplementary References

Table S1. Clinical relevance of cystic fibrosis pathogens with studies featured in the CF-Seq application

Table S1 is available in a separate supplementary file titled "Table S1.xlsx"

Table S2. Applications similar in nature to CF-Seq are described to acknowledge the significant contribution that these researchers have made to making public data more FAIR, and the inspiration their work has provided for CF-Seq. The table also briefly summarizes how these applications are limited for CF pathogen research specifically to demonstrate the unique value of CF-Seq.

Table S2 *is available in a separate supplementary file titled "Table S2.xlsx"*

Table S3. Study Data Acknowledgements. All published data featured in the CF-Seq application is listed in this table, alongside the data contributors cited in GEO and a link to the associated publication, if one exists.

Table S3 *is available in a separate supplementary file titled "Table S3.xlsx"*

Table S4. Example Table of Differentially Expressed Genes. As described in the *A. fumigatus* user story (user story #1), the application was used to compare gene expression for *A. fumigatus* cultured with *P. aeruginosa* vs. *A. fumigatus* cultured alone at a timepoint of 180 minutes. Genes differentially expressed at 180 minutes with p value less than 0.05 were downloaded from the application in a table (514 genes). The resulting list of genes was further filtered to include just those genes with $|log_2FC| > 1.5$ (10 genes, shown here).

Gene ID	log_2FC	log_2CPM	FValue	PValue
CADAFUBG00000399	-1.90	0.44654893	10.5065718	0.00359435
CADAFUBG00004661	2.11	-0.2982177	6.29919199	0.0195418
CADAFUBG00007159	2.15	-0.3214534	6.56882317	0.02079987
CADAFUBG00007744	1.60	1.07297809	4.33649208	0.04858159
CADAFUBG00007752	1.74	-0.0079859	4.9528957	0.0361036
CADAFUBG00007840	-2.04	0.73791847	8.90182797	0.00662776
CADAFUBG00008551	2.05	-0.2801763	5.66923059	0.02847043
CADAFUBG00008638	2.47	0.14680563	4.38436578	0.04745516
CADAFUBG00008721	-1.53	0.29131268	6.20741892	0.02034956
CADAFUBG00009383	1.93	-0.2967983	9.6341365	0.00536067

Table S5. Beta Testing Criteria – Filtering Studies. This table, and the other beta testing tables, we're filled in to identify any bugs in a systematic manner. All identified bugs were subsequently addressed. The app reviewers (three of the paper co-authors, noted in the Contributions section) were also instructed to note suggestions to improve app usability, which were addressed. See the key below the table for species abbreviations (e.g., CA = *Candida albicans*)

Table S5 *is available in a separate supplementary file titled "Table S5.xlsx"*

Table S6. Beta Testing Criteria – Viewing Study Metadata. This table, and the other beta testing tables, we're filled in to identify any bugs in a systematic manner. All identified bugs were subsequently addressed. The app reviewers (three of the paper co-authors, noted in the Contributions section) were also instructed to note suggestions to improve app usability, which were addressed. See the key below the table for species abbreviations (e.g., CA = *Candida albicans*)

Table S6 *is available in a separate supplementary file titled "Table S6.xlsx"*

Table S7. Beta Testing Criteria – Running Analysis. This table, and the other beta testing tables, we're filled in to identify any bugs in a systematic manner. All identified bugs were subsequently addressed. The app reviewers (three of the paper co-authors, noted in the Contributions section) were also instructed to note suggestions to improve app usability, which were addressed. See the key below the table for species abbreviations (e.g., CA = *Candida albicans*)

Table S7 *is available in a separate supplementary file titled "Table S7.xlsx"*

Key (S5-S7): AF = *Aspergillus fumigatus*, Bacteroides = *Bacteroides* species, Burkholderia = *Burkholderia* species, CA = *Candida albicans*, CD = *Clostridium difficile*, FN = *Fusobacterium nucleatum*, HI = *Haemophilus influenza*, MA = *Mycobacterium abscessus*, PA = *Pseudomonas aeruginosa*, Porphyromonas = *Porphyromonas* species, SA = *Staphylococcus aureus*, SM = *Stenotrophomonas maltophilia*, Strep = *Streptococcus* species

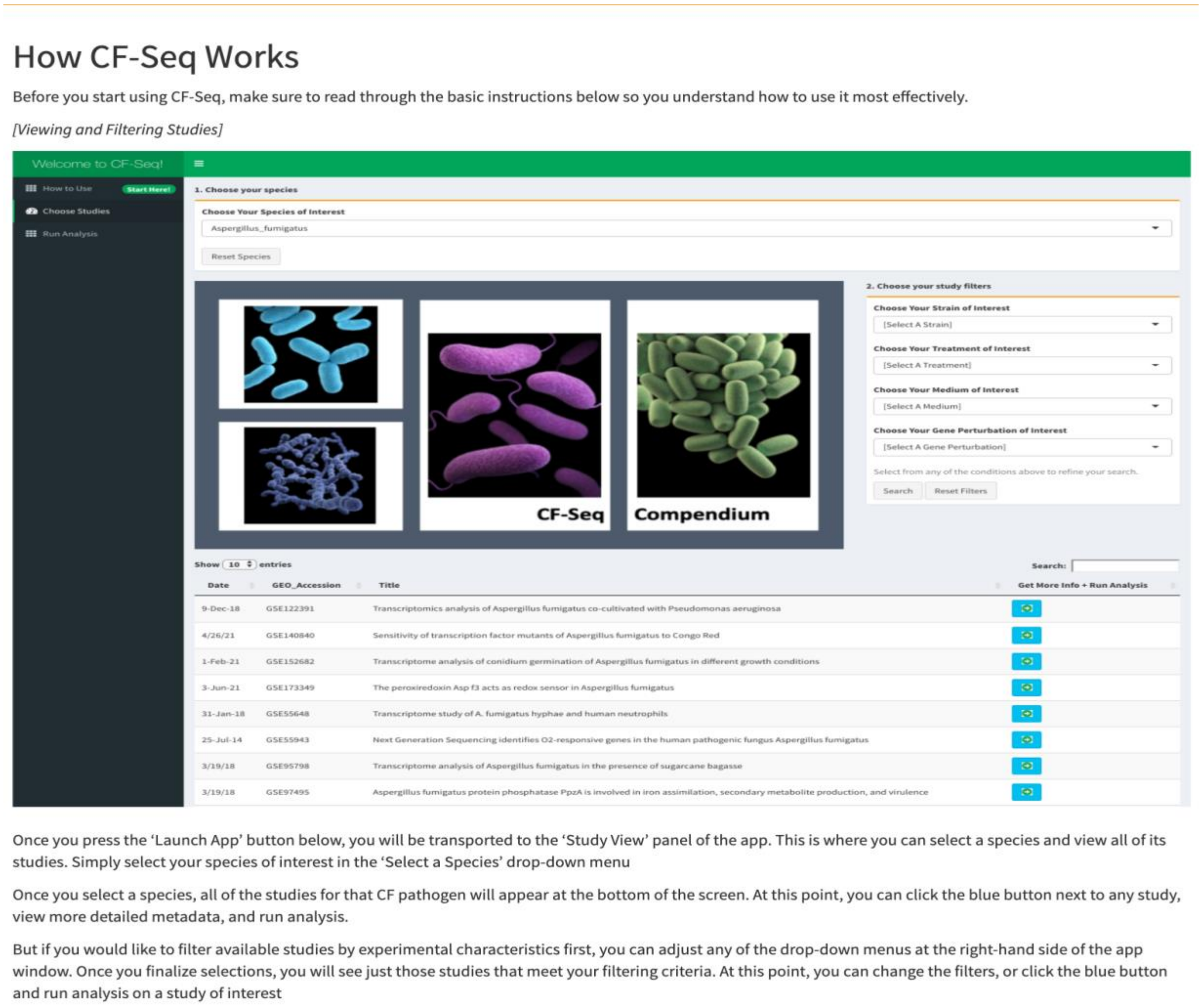

Figure S1. Zoomed-in image of figure 2 (application workflow), panel 1 (user manual)

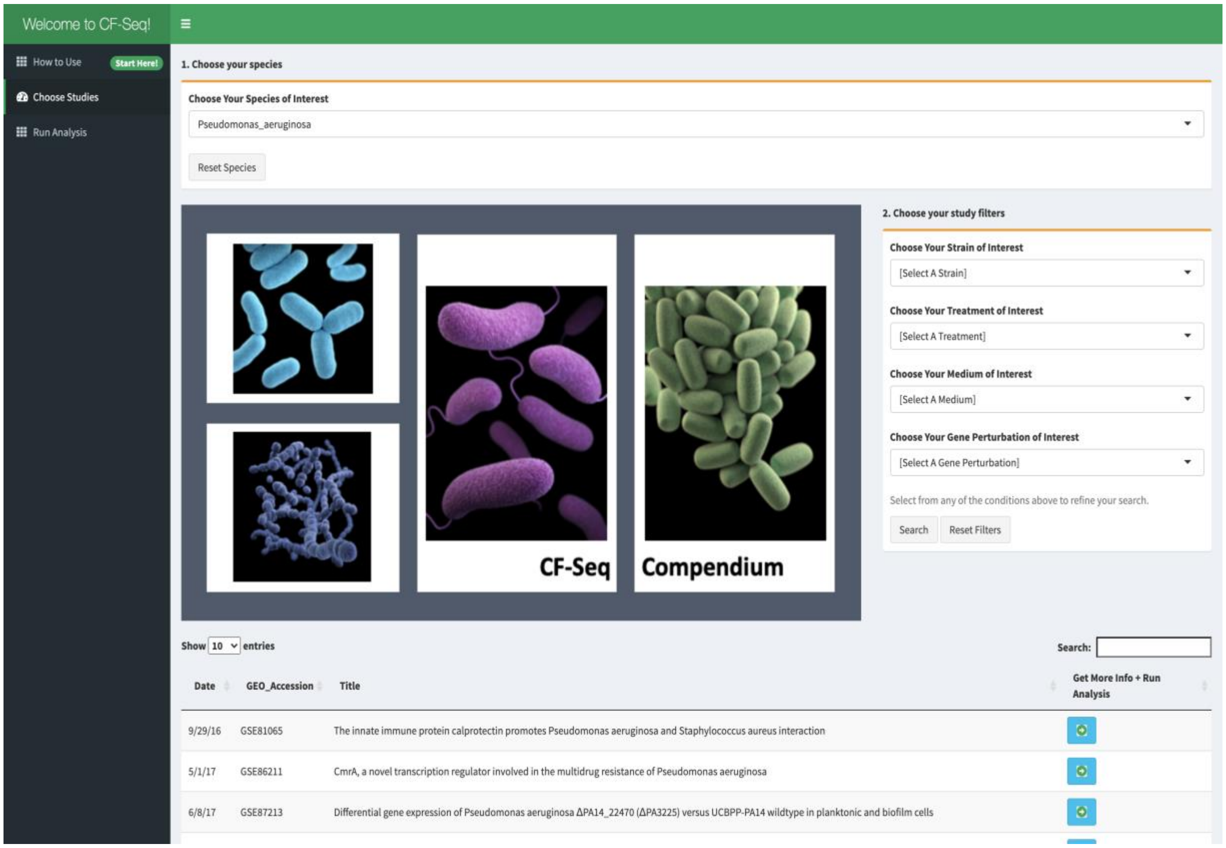

Figure S2. Zoomed-in image of figure 2 (application workflow), panel 2 (study view)

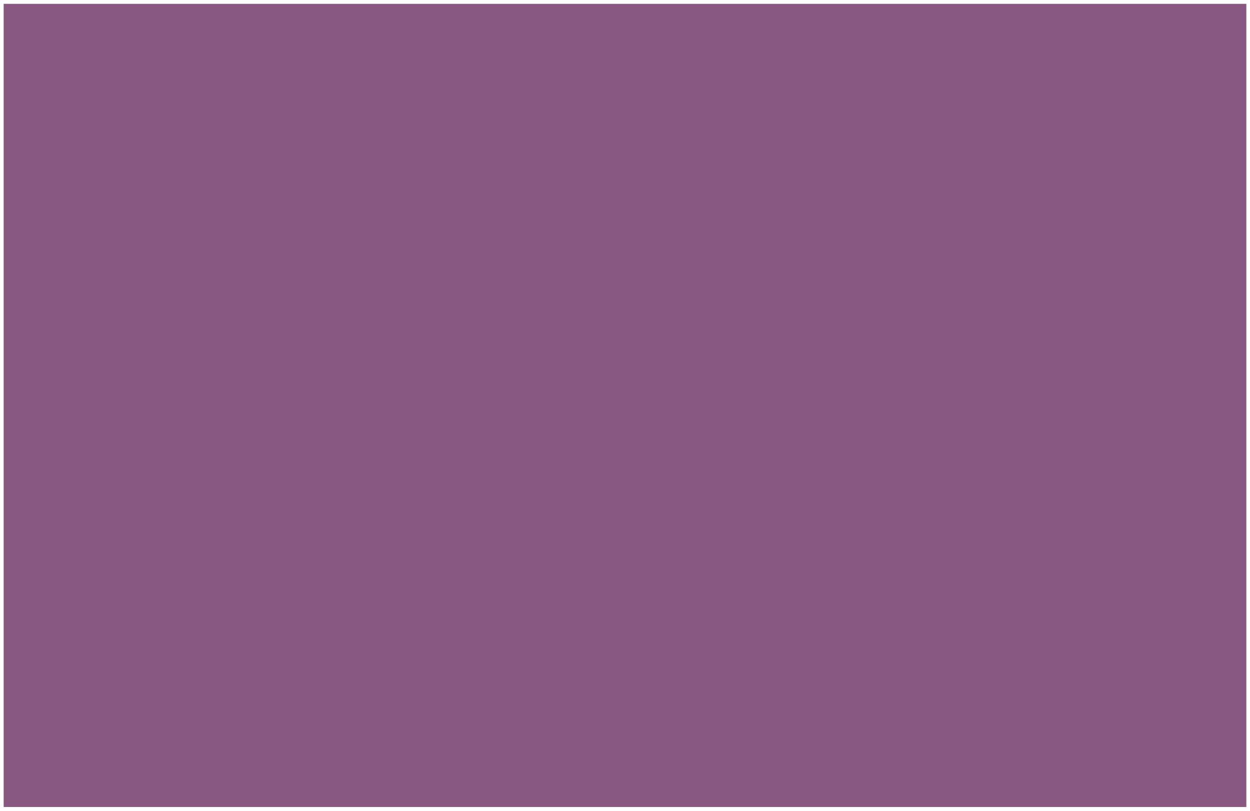

Figure S3. Zoomed-in image of figure 2 (application workflow), panel 3 (filter studies)

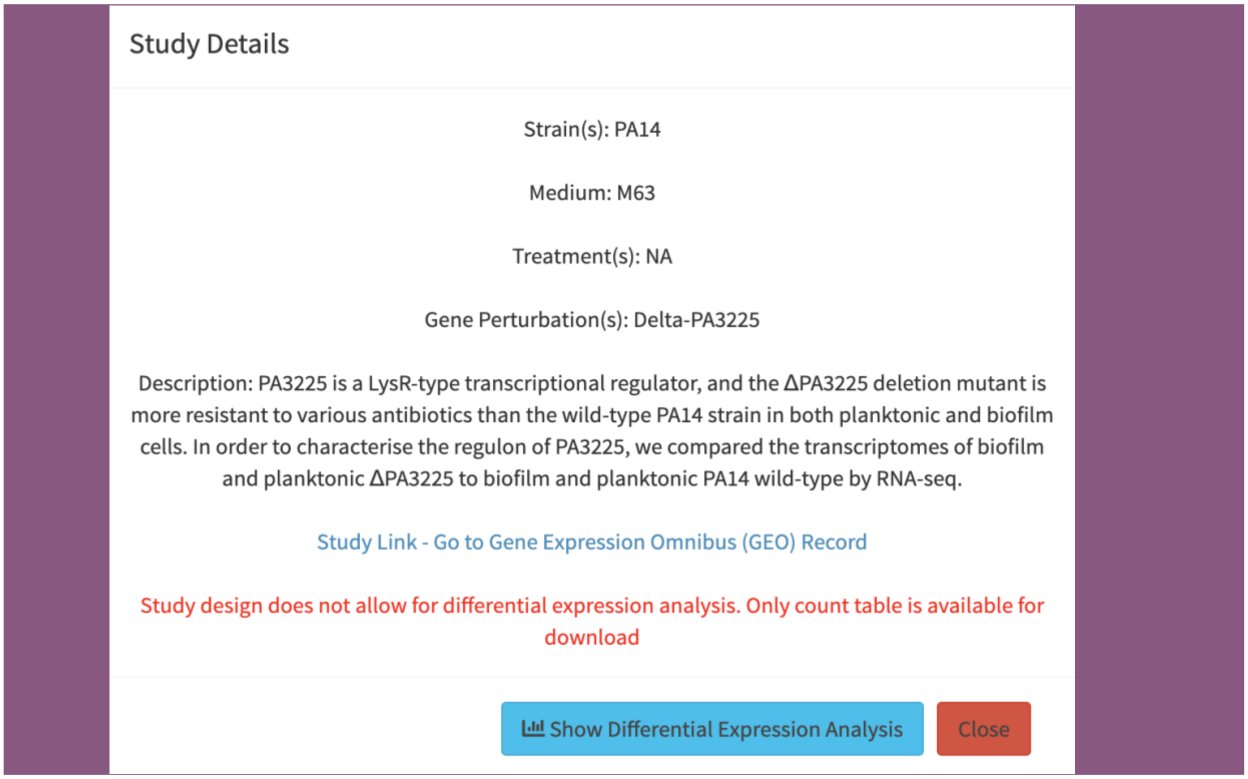

Figure S4. Zoomed-in image of figure 2 (application workflow), panel 4 (additional metadata)

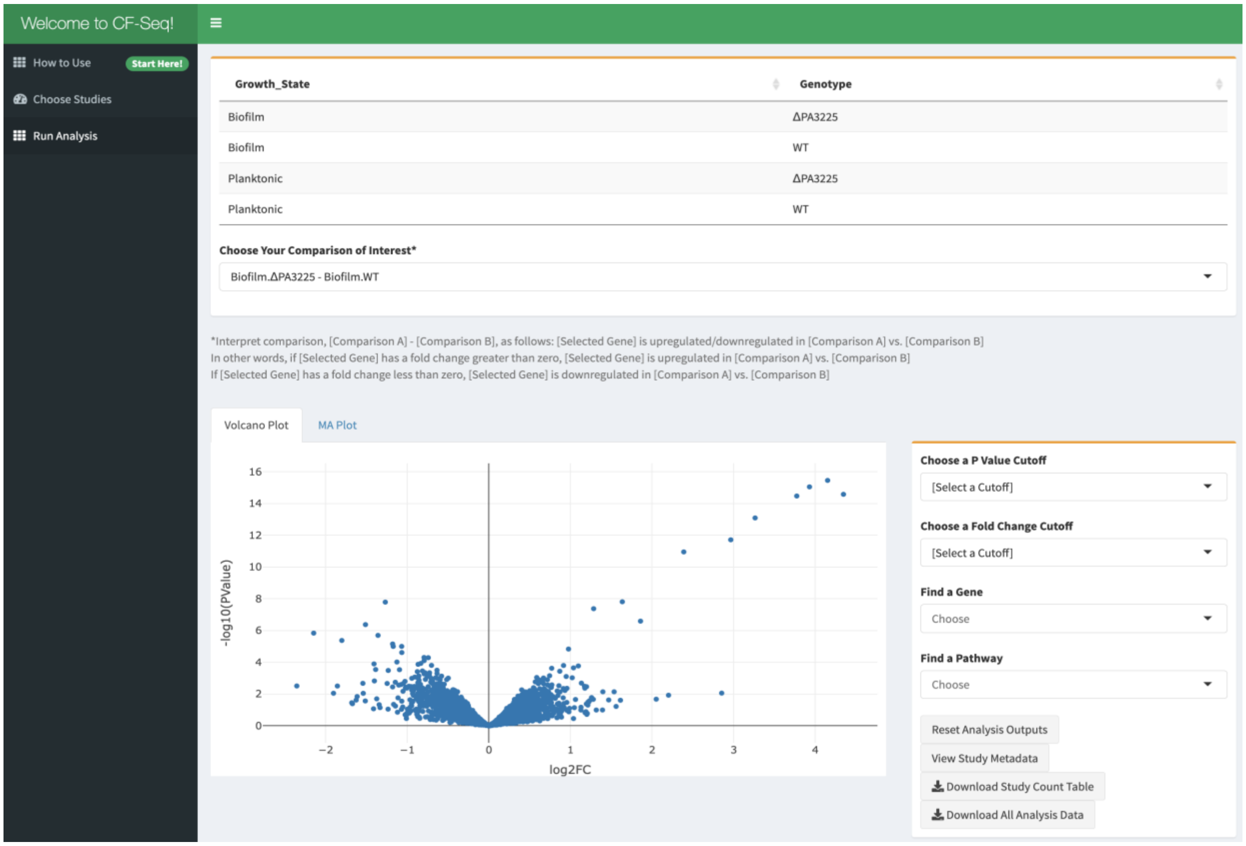

Figure S5. Zoomed-in image of figure 2 (application workflow), panel 5 (analysis view)

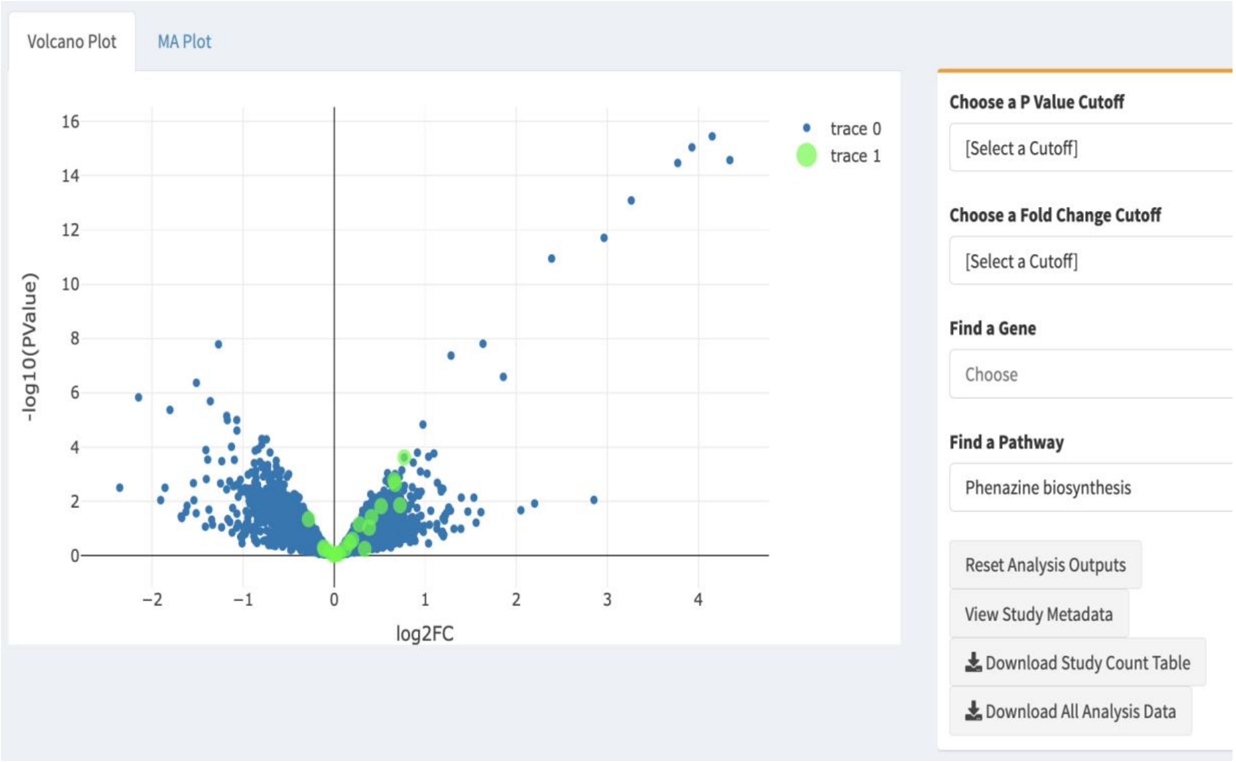

Figure S6. Zoomed-in image of figure 2 (application workflow), panel 6 (visualize pathway)

Supplementary References

The references listed below correspond to the citations in supplementary tables S1 and S2

82. Speirs, J. J., van der Ent, C. K. & Beekman, J. M. Effects of *Aspergillus fumigatus* colonization on lung function in cystic fibrosis. *Current Opinion in Pulmonary Medicine* **18**, 632–638 (2012).
83. Breuer, O. *et al. Aspergillus* Infections and progression of structural lung disease in children with cystic fibrosis. *Am J Respir Crit Care Med* **201**, 688–696 (2020).
84. Lattanzi, C. *et al.* Allergic bronchopulmonary aspergillosis in children with cystic fibrosis: an update on the newest diagnostic tools and therapeutic approaches. *Pathogens* **9**, 716 (2020).
85. Antosca, K. M. *et al.* Altered stool microbiota of infants with cystic fibrosis shows a reduction in genera associated with immune programming from birth. *Journal of Bacteriology* (2019).
86. Duytschaever, G. *et al.* Cross-sectional and longitudinal comparisons of the predominant fecal microbiota compositions of a group of pediatric patients with cystic fibrosis and their healthy siblings. *Applied and Environmental Microbiology* (2011).
87. Zlosnik, J. E. A. *et al. Burkholderia* species infections in patients with cystic fibrosis in British Columbia, Canada. 30 years' experience. *Annals ATS* **12**, 70–78 (2015).
88. Fodor, A. A. *et al.* The adult cystic fibrosis airway microbiota is stable over time and infection type, and highly resilient to antibiotic treatment of exacerbations. *PLOS ONE* **7**, e45001 (2012).
89. Chaparro, C. *et al.* Infection with *Burkholderia cepacia* in cystic fibrosis. *Am J Respir Crit Care Med* **163**, 43–48 (2001).

90. Renner, S., Nachbaur, E., Jaksch, P. & Dehlink, E. Update on respiratory fungal infections in cystic fibrosis lung disease and after lung transplantation. *J Fungi (Basel)* **6**, 381 (2020).

91. Shakirchi, M. A., Klingspor, L., Bergman, P., Hjelte, L. & Monestrol, I. de. A 16-year retrospective study on fungal prevalence and diversity in patients with cystic fibrosis: *Candida dubliniensis* was associated with a decline in lung function. *International Journal of Infectious Diseases* **96**, 663–670 (2020).

92. Piccolo, F. *et al. Clostridium difficile* infection in cystic fibrosis: an uncommon but life-threatening complication. *Respirol Case Rep* **5**, e00204 (2016).

93. Dunwoody, R., Steel, A., Landy, J. & Simmonds, N. *Clostridium difficile* and cystic fibrosis: management strategies and the role of faecal transplantation. *Paediatric Respiratory Reviews* **26**, 16–18 (2018).

94. Mirković, B. *et al.* The role of short-chain fatty acids, produced by anaerobic bacteria, in the cystic fibrosis airway. *Am J Respir Crit Care Med* **192**, 1314–1324 (2015).

95. Li, Q. *et al. Fusobacterium nucleatum* interaction with *Pseudomonas aeruginosa* induces biofilm-associated antibiotic tolerance via *Fusobacterium* Adhesin A. *ACS Infect. Dis.* **6**, 1686–1696 (2020).

96. Hoppe, J. E. & Sagel, S. D. Shifting landscape of airway infection in early cystic fibrosis. *Am J Respir Crit Care Med* **200**, 528–529 (2019).

97. Román, F., Cantón, R., Pérez-Vázquez, M., Baquero, F. & Campos, J. Dynamics of long-term colonization of respiratory tract by *Haemophilus influenzae* in cystic fibrosis patients shows a marked increase in hypermutable strains. *Journal of Clinical Microbiology* (2004).

98. Brugha, R. & Spencer, H. *Mycobacterium abscessus* in cystic fibrosis. *Science* **372**, 465–466 (2021).

99. Ciofu, O. & Tolker-Nielsen, T. Tolerance and resistance of *Pseudomonas aeruginosa* biofilms to antimicrobial agents—how *P. aeruginosa* can escape antibiotics. *Frontiers in Microbiology* **10**, 913 (2019).

100. Malhotra, S., Hayes Jr, D. & Wozniak, D. J. Cystic fibrosis and *Pseudomonas aeruginosa*: the host-microbe interface. *Clinical Microbiology Reviews* (2019).

101. Lamoureux, C. *et al.* An observational study of anaerobic bacteria in cystic fibrosis lung using culture dependant and independent approaches. *Sci Rep* **11**, 6845 (2021).

102. Briaud, P. *et al.* Impact of coexistence phenotype between *Staphylococcus aureus* and *Pseudomonas aeruginosa* isolates on clinical outcomes among cystic fibrosis patients. *Frontiers in Cellular and Infection Microbiology* **10**, 266 (2020).

103. Ahlgren, H. G. *et al.* Clinical outcomes associated with *Staphylococcus aureus* and *Pseudomonas aeruginosa* airway infections in adult cystic fibrosis patients. *BMC Pulmonary Medicine* **15**, 67 (2015).

104. Hansen, C. R. *Stenotrophomonas maltophilia*: to be or not to be a cystic fibrosis pathogen. *Current Opinion in Pulmonary Medicine* **18**, 628–631 (2012).

105. Esposito, A. *et al.* Evolution of *Stenotrophomonas maltophilia* in cystic fibrosis lung over chronic infection: a genomic and phenotypic population Study. *Front Microbiol* **8**, 1590 (2017).

106. Zemanick, E. T. *et al.* Airway microbiota across age and disease spectrum in cystic fibrosis. *Eur Respir J* **50**, 1700832 (2017).

107. Scott, J. E. & O'Toole, G. A. The yin and yang of *Streptococcus* lung infections in cystic fibrosis: a model for studying polymicrobial interactions. *Journal of Bacteriology* (2019).

108. Li, R., Hu, K., Liu, H., Green, M. R. & Zhu, L. J. OneStopRNAseq: a web application for comprehensive and efficient analyses of RNA-seq data. *Genes (Basel)* **11**, 1165 (2020).

109. Nelson, J. W., Sklenar, J., Barnes, A. P. & Minnier, J. The START App: a web-based RNAseq analysis and visualization resource. *Bioinformatics* **33**, 447–449 (2017).

110. Mahi, N. A., Najafabadi, M. F., Pilarczyk, M., Kouril, M. & Medvedovic, M. GREIN: an interactive web platform for re-analyzing GEO RNA-seq data. *Sci Rep* **9**, 7580 (2019).

111. Cheng, X., Yan, J., Liu, Y., Wang, J. & Taubert, S. eVITTA: a web-based visualization and inference toolbox for transcriptome analysis. *Nucleic Acids Research* **49**, W207–W215 (2021).

112. Rinchai, D., Boughorbel, S., Presnell, S., Quinn, C. & Chaussabel, D. A curated compendium of monocyte transcriptome datasets of relevance to human monocyte immunobiology research. (2016).

113. Toro-Domínguez, D. *et al.* ImaGEO: integrative gene expression meta-analysis from GEO database. *Bioinformatics* **35**, 880–882 (2019).
